# Visual evoked potentials of Niemann-Pick type C1 mice reveal an impairment of the visual pathway that is rescued by 2-hydroxypropyl-ß-cyclodextrin

**DOI:** 10.1186/s13023-015-0348-0

**Published:** 2015-10-12

**Authors:** Giampiero Palladino, Stefano Loizzo, Andrea Fortuna, Sonia Canterini, Fioretta Palombi, Robert P. Erickson, Franco Mangia, Maria Teresa Fiorenza

**Affiliations:** Department of Psychology, Section of Neuroscience and “Daniel Bovet” Neurobiology Research Center, Sapienza University of Rome, 00185 Rome, Italy; Department of Therapeutic Research and Medicines Evaluation, Istituto Superiore di Sanità, via Regina Elena 299, 00161 Rome, Italy; Department of Anatomy, Histology, Forensic Medicine and Orthopedics, Unit of Histology and Medical Embryology, Sapienza University of Rome, 00161 Rome, Italy; Department of Pediatrics, University of Arizona, Tucson, AZ 85724-5073 USA

**Keywords:** Lysosomal diseases, Rare disease, *Npc1*, HPßCD, Cholesterol, Neurodegeneration, Dysmyelination

## Abstract

**Background:**

The lysosomal storage disorder, Niemann Pick type C1 (NPC1), presents a variable phenotype including neurovisceral and neurological symptoms. 2-Hydroxypropyl-ß-cyclodextrin (HPßCD)-based therapies are presently the most promising route of intervention. While severe cerebellar dysfunction remains the main disabling feature of NPC1, sensory functions including auditory and olfactory ones are also affected. Morphological and functional anomalies of *Npc1*^*−/−*^ mouse retina have also been observed, although the functional integrity of the visual pathway from retina to visual cortex is still unsettled. We have addressed this issue by characterizing the visual evoked potential (VEP) response of *Npc1*^*−/−*^ mice and determining if/how HPßCD administration influences the VEPs of both *Npc1*^*−/−*^ and *Npc1*^*+/+*^ mice.

**Methods:**

VEP elicited by a brief visual stimulus were recorded from the scalp overlying the visual cortex of adult (PN, postnatal days 60, 75, 85 and 100) *Npc1*^*+/+*^ and *Npc1*^*−/−*^ mice that had received repeated injections of either HPßCD or plain vehicle. The first injection was given at PN4 and was followed by a second one at PN7 and thereafter by weekly injections up to PN49. Cholesterol accumulation and myelin loss were finally assessed by filipin staining and myelin basic protein immunohistochemistry, respectively.

**Results and discussion:**

We have found that the transmission of visual signals from retina to visual cortex is negatively influenced by the loss of *Npc1* function. In fact, the VEP response of *Npc1*^*−/−*^ mice displayed a highly significant increase in the latency compared to that of *Npc1*^*+/+*^ mice. HPßCD administration fully rescued this defect and counteracted the cholesterol accumulation in retinal ganglion cells and dorsal lateral geniculate nucleus neurons, as well as the myelin loss in optic nerve fibers and axons projecting to the visual cortex observed in of *Npc1*^*−/−*^ mice. By contrast, HPßCD administration had no effect on the VEP response of *Npc1*^*+/+*^ mice, further strengthening the treatment efficacy.

**Conclusions:**

This study pinpoints the analysis of VEP response as a potentially accurate and non-invasive approach to assess neural activity and visual information processing in NPC1 patients, as well as for monitoring the progression of the disease and assessing the efficacy of potential therapies.

**Electronic supplementary material:**

The online version of this article (doi:10.1186/s13023-015-0348-0) contains supplementary material, which is available to authorized users.

## Background

Niemann Pick type C1 (NPC1) disease is a rare, inherited lysosomal storage disorder, having variable age of onset and pathophysiological features. The most frequent presentation of the disease is a child of either sex developing coordination problems, dysarthria and hepatosplenomegaly during early school-age years [1, 2]. The disease arises from mutations affecting the function of the protein encoded by the *NPC1* gene that, in cooperation with NPC2, mediates the intracellular trafficking of cholesterol endocytosed via low-density lipoprotein receptors. In fact, NPC1 and NPC2 proteins reside in the membrane and lumen of late endosomes, respectively, and cooperate in the outflow of unesterified cholesterol from late endosomes/lysosomes to endoplasmic reticulum and plasma membrane, thereby enabling its incorporation in various cellular compartments [3, 4]. Defects of either proteins lead to the accumulation of endocytosed unesterified cholesterol and other lipids within lysosomes [5–7]. Approximately 95 % of NPC cases are due to mutations in the *NPC1* gene, and 5 % to mutations in *NPC2* gene [2].

Prominent neurological signs of NPC1 disease in humans, observed also in animal models as *Npc1*^*−/−*^ mice and cats, are cerebellar dysfunctions landmarked by the progressive degeneration of cerebellar Purkinje cells [8, 9] leading to ataxia [10]. However, other brain areas are also involved in the disease, reducing the efficiency of the processing of sensory information, including auditory, olfactory and visual signals [11]. As indicated by studies in the well established NPC1 mouse model, *Npc1*^*nih/nih*^ (*Npc1*^*−/−*^) [12], these defects are likely associated with severe neuronal and glial damage in brain areas as the thalamus, which act as relay stations for sensory information on the way to the cortex, whereas neurons of other brain regions, including the cerebral cortex, appear less vulnerable to NPC1-deficiency [13–15]. Within the thalamus of *Npc1*^−/−^ mouse, significant neuronal loss and marked gliosis are detected in ventral postero-medial/lateral (VPM/VPL, somatosensory), dorsal lateral geniculate (dLGN, visual) and medial geniculate (MGN, auditory) nuclei as early as at 3 weeks of age [14]. Interestingly, hearing loss is among neurological signs frequently associated with NPC1 disease in our species [2, 16] and appears to rely on auditory brainstem neuropathology [17]. In fact, typical features of lysosomal cholesterol accumulation have been observed in the cochlea, inferior colliculus and MGN of *Npc1*^−/−^ mice [18], which also display abnormal auditory brainstem responses as early as at postnatal day 20 (PN20) [17].

Besides the auditory pathway, the cellular dysfunctions of *Npc1*^−/−^ mouse retina and dLGN suggest that the visual pathway is also affected in NPC1 disease. Morphological and functional anomalies of the retina of these mice were recently reported, showing age-related degeneration of photoreceptors and a number of anomalies in several cell types of the visual system, including increased autophagy in retinal ganglion cell layer (GCL), electron-dense inclusions in bipolar and Müller cells, abnormal arborization of horizontal and amacrine cells, altered myelination, dilated axons of the optic nerve [19] and reduced amplitude of electroretinogram (ERG) responses [20]. However, studies investigating the functional integrity of the visual pathway from retina to visual cortex are still lacking.

Visual evoked potentials (VEPs) are a well established tool for assessing modifications of the visual pathway integrity and visual cortex functioning during normal aging and neurodegeneration in both humans and animal models [21–24]. Therefore, in this study we have exploited VEP recording to investigate whether visual signal transmission from retina to visual cortex is impaired in *Npc1*^*−/−*^ mice. The preliminary evidence that the visual pathway is actually defective in these mice prompted us to also assess the rescuing efficacy of 2-hydroxypropyl-ß-cyclodextrin (HPßCD), a drug representing the major treatment currently studied in both patients and animal models of NPC1 disease.

The use of HPßCD was pioneered in a NPC1 mouse model by Camargo et al. [25], but it was only when this drug was administered earlier in life and at higher doses that its therapeutic efficacy was widely appreciated [26–28]. In fact, extensive efforts using *Npc1*^*−/−*^ mouse and cat models have thoroughly demonstrated the ability of HPßCD to mobilize intracellular cholesterol [29–34], leading to a phase I clinical trial that started in 2013 [35]. However, while HPßCD is considered generally safe, recent studies have shown that it may cause dose-dependent hearing loss in normal mice and in cats affected by NPC disease [33, 36]. In light of this warning on the safety of HPßCD treatment, the experimental design of this study included the treatment of both *Npc1*^*+/+*^ and *Npc1*^*−/−*^ littermates.

Our results show that the visual stimulus transmission from retina to visual cortex is significantly delayed in *Npc1*^*−/−*^ mice compared to age-matched *Npc1*^*+/+*^ and that HPßCD administration rescues this defect, having no apparent effect on *Npc1*^*+/+*^ mice.

## Methods

### Animals and treatments

**Fig. 1:**
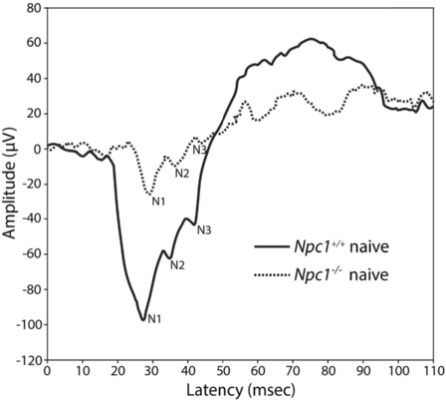
VEP components of naïve PN75 *Npc1* ^*+/+*^ and *Npc1* ^*−/−*^ mice. Representative VEPs are shown in the figure. Note the increase in N1, N2 and N3 latency and the reduction in amplitude displayed by *Npc1* ^*−/−*^ compared to *Npc1* ^*+/+*^ mice. N1, first negative peak; N2, second negative peak; N3, third negative peak

*Npc1*^*−/−*^ mice are maintained on BALB/cJ background and are derived from heterozygous matings. However, instead of the expected 25 %, only 17 % of *Npc1*^*−/−*^ are weaned. Genotypes were identified at weaning by PCR on tail DNA as described [12]. HPßCD administration was performed by subcutaneous injection of either a 20 % solution (w/v; 4000 mg/Kg body weight) of HPßCD (average degree of substitution of 0.67 of hydroxypropyl groups per glucose unit, MW ~1369 Da, catalog number H-107, Sigma Aldrich, Milan, Italy) in PBS, or plain PBS as control. The treatment schedule was as follows: both *Npc1*^*+/+*^ and *Npc1*^*−/−*^ pups received two consecutive subcutaneous injections at PN4 and PN7, followed by weekly injections until PN49. Age-matched control mice received plain PBS injections (sham animals). Within the following 1–3 days (PN50-52) mice were chronically implanted with electrodes. For HPßCD/sham administration, pre-weaning PN4-PN21 pups were separated from the mother, weighed, injected at the scruff of the neck [26], monitored for complete absorption of the injection bolus and finally returned to the home cage. The entire injection procedure lasted approximately 15–20 min, giving each pup a similar treatment, inclusive of maternal separation, handling and injection. A scheme summarizing the schedule of injection, electrode implantation and VEP recording is shown in (Additional file 1: Figure S1). HPßCD administration according to the schedule described above significantly increased the life expectancy of *Npc1*^*−/−*^ mice (Additional file 2: Figure S2). Given that heterozygous matings limited the availability of *Npc1*^*−/−*^ and *Npc1*^*+/+*^ pups, experimental groups were formed and processed as follows: a first group of *Npc1*^*+/+*^ (*n* = 12) and *Npc1*^*−/−*^ (*n* = 12) age-matched mice that had not received any kind of handling before electrode implantation (naive mice) were subjected to VEP recordings at PN75 (Fig. 1); a second group of *Npc1*^*+/+*^ and *Npc1*^*−/−*^ age-matched mice, either HPßCD-treated or sham (*n* = 13 in each experimental group), were subjected to VEP recordings at PN60, PN75, PN85 and PN100, except for 5 mice of each experimental group that were sacrificed immediately after the PN75 recording for histological analyses. In addition, the *Npc1*^*−/−*^ mice were never recorded at PN100 because they did not survive after PN90-PN95. All mice were maintained in our animal facility in accordance with Sapienza University guidelines for the care and use of laboratory animals. Experimental protocols and related procedures were approved by the Italian Ministry of Public Health.

### Visual Evoked Potential (VEP) recording

VEP experiments were performed according to published procedures [37, 38]. Briefly, mice were anaesthetized by intraperitoneal injection of a mixture of xylazine (Rompun, Bayer AG, Leverkusen-Germania, 20 mg/Kg i.p) and ketamine (Ketavet 100, Gellini Farmaceutici Spa, Peschira Borromea-MI, 32 mg/Kg i.p.) and locally injected with lidocaine (xylocaine 2 %, 0.1 ml under the scalp) before the implantation of chronic electrodes. Three epidural L-shaped monopolar stainless steel electrodes were implanted (right anterior: 1.3 mm anterior to bregma and 1.5 mm lateral to sagittal suture; right and left posterior: 3 mm posterior to bregma and 2.5 lateral to sagittal suture), with the right posterior (active) electrode position within the area of maximal visual cortex acuity [39]. Electrophysiological recording started 1 week after electrode implantation. Each mouse, awake and free to move, was dark-adapted for 20 min in an anechoic testing cage placed in a dark, soundproofed and electrically-shielded room before VEP recording. Light flash-VEPs were elicited by a Grass-Instruments PS22 photic stimulator (stimulus intensity 236.4 cd/m^2^/s; stimulus rate 1 flash/s; stimulus duration 10 μsec) located outside the sound-proofed cage to avoid acoustic contamination from the strobe. To avoid possible interferences due to circadian rhythm, VEPs were always recorded between 09:00 and 12:00 a.m., EEG signals were amplified through a DC-powered preamplifier with a gain of 1000× and an analogic high-pass filter at 1 Hz and a low-pass filter at 10 kHz. Acquisitions were performed at 2.5 kHz sampling rate. An InstruNet A/D 16 bit conversion board delivered signals to the acquisition system (Superscope GW Instruments, Somerville, Massachusetts, USA, adapted by Analysa, Cuneo, Italy) on a personal computer. Brain activity and trigger were continuously acquired and saved as raw data to be analyzed off-line. VEPs from a sequence of 90 strobe light flash were averaged to obtain the final waveform. After a pause of 5 min, the same mouse was again stimulated by another series of 90 stimuli and this procedure was repeated for a total of 6–8 times in the same day. Registrations made anomalous by the occurrence of artifacts and/or the insurgence of seizures, as previously described in *Npc1*^*−/−*^ mice [40], were discarded. VEP amplitude and latency were measured after baseline removal. N1, N2 and N3 latencies were calculated as the times elapsed between the onset of first stimulus and the peak of the first, second and third negative responses, respectively, whereas N1 amplitude was taken as the absolute value of the first negative peak. Each mouse was used for repeated sessions at increasing ages (PN58-62, PN73-77, PN83-87, PN98-102), here for brevity indicated as PN60, PN75, PN85, PN100, except for the *Npc1*^*−/−*^ mice that, if not treated with HPßCD, were not analyzed after PN85 because they usually died at PN90-95 days, as previously reported [31].

### Detection of intracellular cholesterol and myelin

PN75 *Npc1*^+/+^ and *Npc1*^*−/−*^ mice*,* either injected with HPßCD or sham according to the schedule of Additional file 1: Figure S1, were anaesthetized by intraperitoneal injection of a mixture of xylazine/ketamine (20–34 mg/kg i.p) and then transcardially perfused with 4 % paraformaldehyde (PFA) in PBS. For filipin staining, brains, eyes and optic nerves were removed, post-fixed overnight at 4 °C in 4 % PFA, cryoprotected with sucrose (30 % in PBS), embedded in frozen section compound (FSC22 Clear^R^ Frozen Section Compound, Leica Biosystems, Milan, Italy) and serially sectioned (slice thickness 10 μm) using a cryostat (Leica CM 1900). Sagittal sections were mounted on adhesive glass slides (X-tra glass slides, Leica Biosystems), washed in PBS and post-fixed in cold acetone at −20 °C for 10 min and then in 3 % PFA for 10 min. Sections were incubated overnight at 4 °C in a buffer made of 0.1 M glycine in PBS supplemented with 0.3 % BSA, stained with filipin (10 μg/ml in PBS) for 3 h at room temperature and finally mounted with buffered glycerol. For myelin basic protein (MBP) detection, PFA-fixed tissues were dehydrated, embedded in Paraplast Tissue Embedding Medium (Leica Biosystems) and serially sectioned (slice thickness 8 μm). Sagittal sections were mounted on X-tra Adhesive glass slides (Leica Biosystems), de-waxed with xylene, re-hydrated and washed in PBS. Epitopes were unmasked by heating sections twice (10 min each) in 0.1 M citric acid, pH 6.0, in a microwave oven. After washing with PBS, sections were incubated overnight at 4 °C with a monoclonal anti-MBP antibody (Sigma-Aldrich, Milano, Italy; 1:100 dilution in PBS), and then stained using the rabbit Vectastain Elite ABC Kit (Vector Laboratories Inc., Burlingame, CA, USA), followed by the DAB Peroxidase Substrate Kit (Vector Laboratories Inc.) according to manufacturer’s instructions. Epifluorescent images of filipin/MBP staining (Additional file 3: Figure S3) were obtained using a Zeiss Axioplan fluorescent microscope equipped with Cool Snap K4 Photometrics camera or a Sony nex-3 N mirrorless camera (Sony Europe Limited, Milano, Italy). Images were processed using ImageJ NIH software version 1.48v.

### Gross histology and cell counts

The cytoarchitecture and cell number of visual cortex and dLGN were determined by staining sagittal sections of PN75 *Npc1*^*+/+*^ and *Npc1*^*−/−*^, either sham or HPßCD-treated, tissues with hematoxylin (Sigma-Aldrich) for 15 min and then eosin Y (0.3 % in ethanol 80 %; Sigma-Aldrich) for 15 s. The counting of cell nuclei was performed on three consecutive sagittal sections of at least five mice/group, using the function “cell counter” of ImageJ NIH software. Three region of interest (ROI, 400 μm^2^ each) were randomly selected in each field as previously described [41], and histological observations and evaluations were blindly and independently performed by two investigators.

### Statistics

Statistical analyses were performed using GraphPad Prism version 5.0d (GraphPad, La Jolla, CA). Data were tested for normality (Wilk-Shapiro’s test) and homoscedasticity (Levene’s test) and then analyzed by unpaired two-tailed Student’s t test, one-way ANOVA, two-way ANOVA for independent (group, genotype, treatment) measures, and repeated measures one-way ANOVA followed by Tukey’s HSD post-hoc test. *P* values smaller than 0.05 were considered significant.

## Results

### An abnormal VEP indicates a decreased visual pathway efficiency of *Npc1*^*−/−*^ mice

VEPs consist of electrical potentials elicited by a brief visual stimulus that are recorded from the scalp overlying the visual cortex. Typically, VEP waveforms consist of a sequence of voltage deflections named peaks, waves or components. The shape of representative VEPs of *Npc1*^*+/+*^ and *Npc1*^*−/−*^ adult (PN75) mice is displayed in Fig. 1. The three negative VEP components, N1, N2 and N3, showed that the VEP response is strongly influenced by *Npc1* deficiency. In fact, N1-N3 VEP components of *Npc1*^*−/−*^ mice appeared with a delay of 1.5–2 msec (latency) and displayed a reduced voltage amplitude compared to those of *Npc1*^*+/+*^ mice. The values of VEP latency and amplitude recorded in this experiment are reported in Table 1.

**Table 1:** Effect of genotype on VEP latency and amplitude in naive PN75 *Npc1* ^*+/+*^ and *Npc1* ^*−/−*^ mice^a^

	*Npc1* ^*+/+*^ (*n* = 12)	*Npc1* ^*−/−*^ (*n* = 12)	*t*	*(df)* ^*b*^	*p* ^*c*^
Latency, N1	27.59 ± 0.16^d^	28.98 ± 0.59	2.29	(22)	0.0396
Latency, N2	35.08 ± 0.62	37.35 ± 0.55	2.75	(22)	0.0118
Latency, N3	43.37 ± 0.81	44.70 ± 0.85	1.14	(22)	0.2778
Amplitude, N1	105.20 ± 13.67	22.00 ± 6.32	5.52	(22)	0.0001

^a^The mice examined in this experiment did not receive any kind of experimental manipulation before electrode implants and VEP recording

^b^Degree of freedom

^c^ *p* was evaluated by unpaired two-tailed Student’s *t* test

^d^mean ± SEM

### VEP recordings of HPßCD-treated *Npc1*^*+/+*^ and *Npc1*^*−/−*^ mice display a specific rescue of VEP latency in *Npc1*^*−/−*^ mice

Based on the defective VEP responses of *Npc1*^*−/−*^ mice, we settled to investigate the efficacy of HPßCD administration in rescuing this defect. Studies aimed at characterizing the ability of HPßCD to circumvent defective cholesterol trafficking and disease severity in *Npc1*^*−/−*^ mice have so far exploited either a single injection at PN7 [27] or repeated injections starting at PN7 or at weaning [25, 28, 31], leading to the conclusion that earlier injections resulted in a stronger efficacy. Therefore, to boost the efficacy of HPßCD treatment, we designed a treatment schedule consisting of two subsequent HPßCD injections at PN4 and PN7, namely before the blood brain barrier is completely formed [42] and when cholesterol synthesis in the mouse brain reaches its highest level [43], making it reasonable the expectation that the neuronal import of glia-derived cholesterol is maximal. We also sought to continue injections after PN7 by weekly injecting mice up to 7 weeks of age (see Additional file 1: Figure S1).

**Fig. 2:**
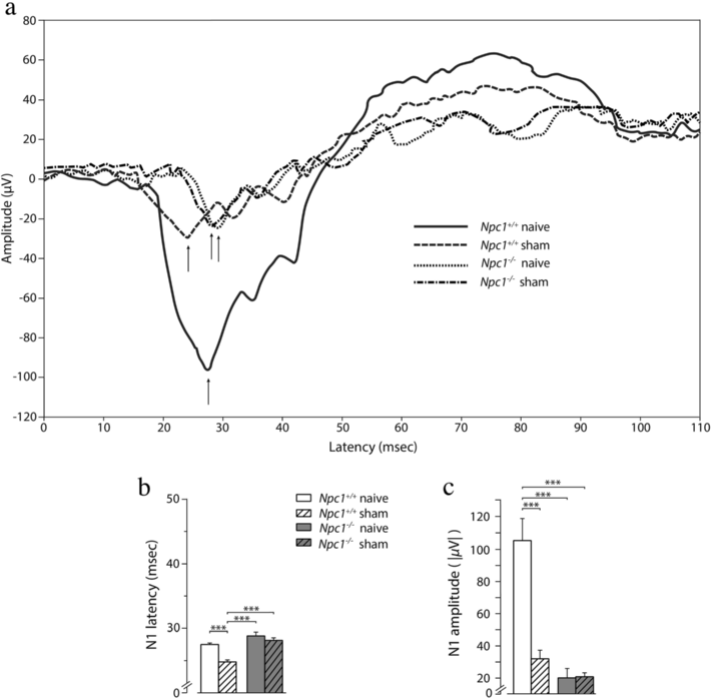
VEP components of naïve/sham PN75 *Npc1* ^*+/+*^ and *Npc1* ^*−/−*^ mice. **a** Representative VEPs are shown in panel a (see Fig. 1 for description). Note the different values of N1 peak latency and amplitude (*arrows*) depending on experimental manipulation and/or genotype. **b, c** Histograms indicate the mean ± SEM of N1 latency and amplitude values obtained in the experimental groups indicated in the panels. Note that the latency of sham *Npc1* ^*+/+*^ mice significantly decreased compared to that of naïve *Npc1* ^*+/+*^ mice, whereas sham and naïve *Npc1* ^*−/−*^ mice had similar latency. The amplitude of sham mice, either *Npc1* ^*+/+*^ or *Npc1* ^*−/−*^ *,* did not significantly differ from each other, but were significantly reduced compared to that of *Npc1* ^*+/+*^ naïve mice. Asterisks indicate statistically significant differences among experimental groups (***, *p* < 0.001)

Because of the novelty introduced in our protocol, with particular reference to the early injection at PN4 shortly followed by an additional injection at PN7, we sought to preliminarily evaluate the effect of plain PBS, i.e. the vehicle of HPßCD administration (see Methods), by comparing the VEP recordings of naïve *Npc1*^*+/+*^ and *Npc1*^*−/−*^ mice of Fig. 1 with representative VEP recording of sham mice (Fig. 2a). Interestingly, the injections of plain PBS significantly influenced the VEP response of *Npc1*^*+/+*^ mice. In fact (Fig. 2b) these *Npc1*^*+/+*^ mice (thereafter named “sham”) displayed a significantly shortening of N1 latency (24.77 ± 0.32 msec) compared to that of naive *Npc1*^*+/+*^ mice (27.59 ± 0.16 msec). By contrast, the latency of PN75 sham *Npc1*^*−/−*^ was similar to that of naive *Npc1*^*−/−*^ mice (28.16 ± 0.40 msec and 28.98 ± 0.59 msec, respectively). We also noticed that the VEP amplitudes of sham mice, either *Npc1*^*+/+*^ or *Npc1*^*−/−*^*,* did not significantly differ from each other (N1: 32.36 ± 5.32 μV and 20.91 ± 2.22 μV, respectively; *p* < 0.05), in spite of the significant difference in N1 amplitude values of naive *Npc1*^*+/+*^ and *Npc1*^*−/−*^ mice (Fig. 2c). The complete output of statistical analyses of VEP N1 latency and amplitude values is reported in (Additional file 4: Table S1), whereas significant differences of post-hoc comparisons are indicated by asterisks in Fig. 2.

**Fig. 3:**
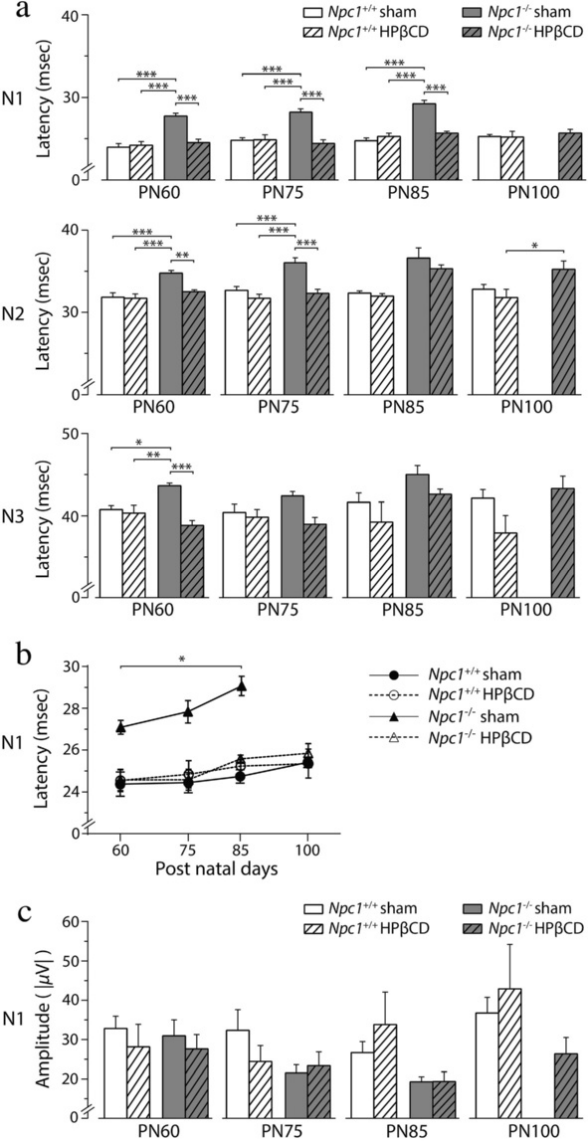
VEP latency and amplitude in PN60, PN75, PN85 and PN100, either sham or HPßCD-treated, *Npc1* ^*+/+*^ and *Npc1* ^*−/−*^ mice. **a** Histograms indicate the mean ± SEM of N1, N2, and N3 latency values obtained in different experimental groups at increasing ages, as indicated in the panel. Note that HPßCD fully rescued the latency increase observed in *Npc1* ^*−/−*^ mice, whereas it had no effect on *Npc1* ^*+/+*^ mice. PN100 sham *Npc1* ^*−/−*^ mice are not reported because these animals never survived beyond PN95, unless treated with HPßCD. **b** Effect of the age on N1 latency of various experimental group. Note that the latency significantly increased with age in sham *Npc1* ^*−/−*^ mice, but not in *Npc1* ^*+/+*^ mice, and that such increase was effectively prevented by treatment with HPßCD (*, *p* < 0.05). **c** Histograms indicate the mean ± SEM of N1 amplitude values obtained in different experimental groups at increasing ages, as indicated in the panel. Asterisks indicate statistically significant differences among experimental groups (*, *p* < 0.05; **, *p* < 0.01; ***, *p* < 0.001)

In light of the observations reported above, subsequent determinations of the effect of HPßCD on *Npc1*^*+/+*^ and *Npc1*^*−/−*^ mice, thereafter named “HPßCD”, were performed using age-matched sham mice as controls. These determinations provided a wealth of information that is summarized as follows. First (Fig. 3a, b), the N1 latencies of both sham and HPßCD-treated *Npc1*^*+/+*^ mice were similar with each other (24.77 ± 0.32 msec and 24.82 ± 0.60 msec, respectively; *p* > 0.05), but significantly shorter (*p* < 0.05) than that of *Npc1*^*+/+*^ naïve mice. Similar results were also obtained for N2 and N3 latencies. We therefore concluded that HPßCD and PBS injections had similar effects on *Npc1*^*+/+*^ mice, indicating a dependence on the injection and/or maternal separation *per se.* Second, HPßCD administration had a beneficial and highly specific effect in normalizing the VEP latency of *Npc1*^*−/−*^ mice. Indeed, the latency of N1 wave appeared significantly earlier in HPßCD-treated *Npc1*^*−/−*^ compared to that of *Npc1*^*−/−*^ sham mice (24.39 ± 0.45 msec and 28.16 ± 0.40 msec, respectively; *p* < 0.05), indicating that this effect depended on the HPßCD administration rather than on the injection and/or maternal separation. Moreover, the N1 latency values of HPßCD-treated *Npc1*^*−/−*^ mice were similar to those of either HPßCD or sham *Npc1*^*+/+*^ mice (24.82 ± 0.60 msec and 24.77 ± 0.32 msec, respectively; *p* > 0.05), indicating that HPßCD effectively rescued cellular/molecular substrates underlying the *Npc1*^*−/−*^ deficiency. Similar results were also obtained for N2 and N3 latencies. It is also interesting to note that the N1 latency of sham *Npc1*^*+/+*^, HPßCD-treated *Npc1*^*+/+*^ and HPßCD-treated *Npc1*^*−/−*^ mice did not increase from PN60 to PN85, whereas it significantly increased in sham *Npc1*^*−/−*^ mice (Fig. 2b), suggesting that HPßCD effectively prevented the visual system impairment that *Npc1*^*−/−*^ mice undergo with aging. The statistical analysis of this panel is reported in (Additional file 5: Table S2).

In contrast to latency, VEP amplitude (Fig. 3c) was not significantly affected by HPßCD in both *Npc1*^*−/−*^ and *Npc1*^*+/+*^ mice, indicating that the observed variations in this parameter were fully independent on the drug. The complete output of statistical analyses of VEP N1, N2, N3 latency and N1 amplitude values recorded at PN60, PN75, PN85, PN100 are reported in (Additional file 6: Table S3, Additional file 7: Table S4), whereas significant differences of post-hoc comparisons are indicated by asterisks in Fig. 3.

### HPßCD rescues the abnormal cholesterol accumulation of *Npc1*^*−/−*^ mice

**Fig. 4:**
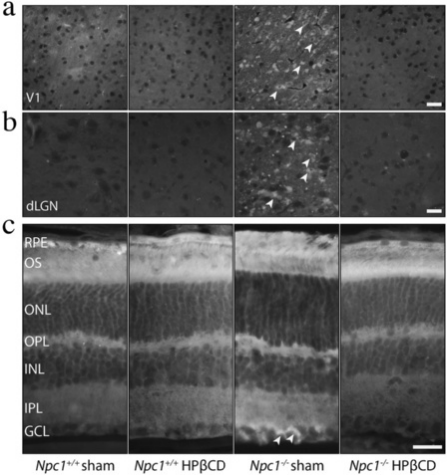
HPßCD corrects the cholesterol storage defect of *Npc1* ^*−/−*^ mice. Brain parasagittal sections of PN75 *Npc1* ^*+/+*^ and *Npc1* ^*−/−*^ mice, either sham or HPßCD-treated, were stained with filipin. Representative fields (Additional file 3: Figure S3) of **a** primary visual cortex (V1), **b** dorsal lateral geniculate nucleus (dLGN) and **c** retinal ganglion cells are shown in the figure. Note that the cytoplasmic cholesterol accumulation (*arrowheads*) is almost fully rescued by HPßCD. RPE: retinal pigment ephitelium; OS: outer segments; ONL: outer nuclear layer; OPL: outer plexiform layer; INL: inner nuclear layer; IPL: inner plexiform layer; GCL: ganglion cell layer. Scale bars indicate 50 μm (**a**, **b**) and 25 μm (**c**)

To verify whether HPßCD not only normalized the VEP latency of *Npc1*^*−/−*^ mice, but also rescued the abnormal cellular features typical of the disease, we visualized intracellular cholesterol accumulation by filipin staining on brain slices of PN75 sham or HPBCD-treated, *Npc1*^*+/+*^ and *Npc1*^*−/−*^ mice. We first focused this analysis on visual cortex and thalamic dLGN (representing the only relay station for visual information on its way from retina to visual cortex) [44, 45]. Interestingly, the HPßCD administration almost completely eliminated the filipin-stained cholesterol deposits present in the visual cortex and dLGN of *Npc1*^*−/−*^ sham mice (Fig. 4a, b). Similar features were also observed in retinal ganglion cells (Fig. 4c), whereas HPßCD administration did not have any effect on *Npc1*^*+/+*^ mice (Fig. 4a–c).

### HPßCD rescues the myelin loss of *Npc1*^*−/−*^ mice

**Fig. 5:**
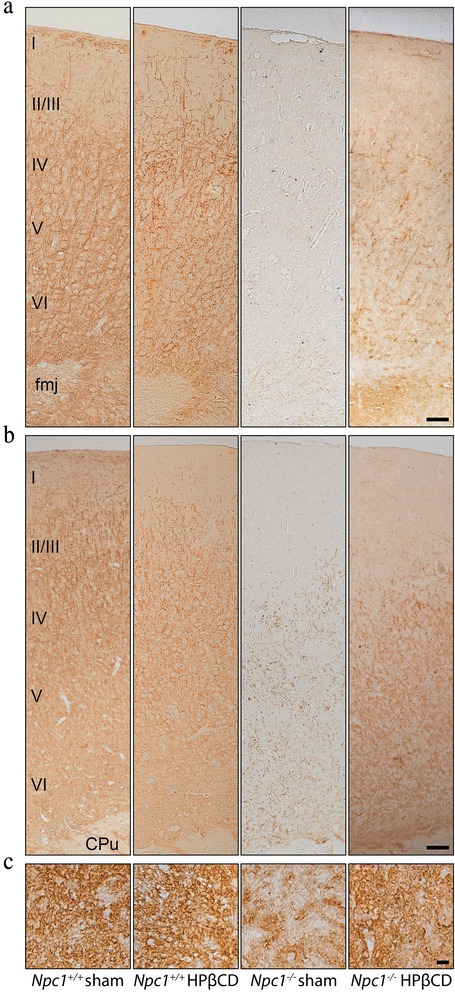
HPßCD counteracts the myelin loss of *Npc1* ^*−/−*^ mice. Brain sections of PN75, either sham or HPßCD-treated, *Npc1* ^*+/+*^ and *Npc1* ^*−/−*^ mice were immunostained with anti-myelin basic protein antibody (brown). Representative fields (see Additional file 3: Figure S3) of parasagittal sections of **a** primary visual cortex **b** primary somatosensory cortex, and **c** transversal sections of optic nerve are shown in the figure. The robust myelin reduction of *Npc1* ^*−/−*^ mice was partially rescued by HPßCD administration. I–VI: cortical layers; fmj: forceps major corpus callosum; CPu: caudate putamen (striatum). Scale bars indicate 50 μm (**a**, **b**) and 6 μm (**c**)

**Fig. 6:**
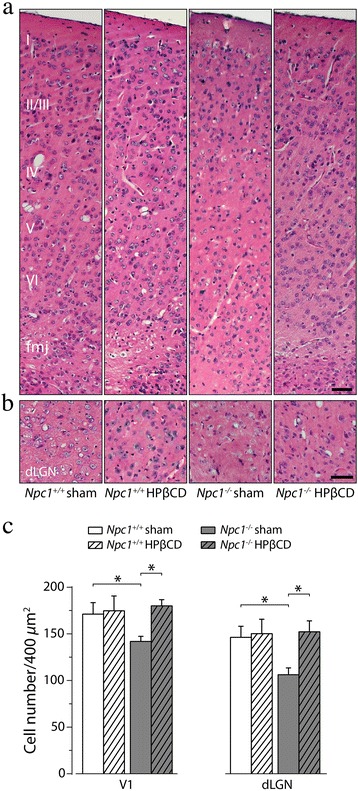
HPßCD rescues the reduced cell number of *Npc1* ^*−/−*^ mice. Brain sections of PN75, either sham or HPßCD-treated, *Npc1* ^*+/+*^ and *Npc1* ^*−/−*^ mice were stained with haematoxylin and eosin. Representative fields (see Additional file 3: Figure S3) of **a** primary visual cortex and **b** dorsal lateral geniculate nucleus (dLGN) are shown in the figure. **c** Histograms indicate cell densities (mean ± SEM) determined on 5 randomly selected 400 μm^2^ regions of primary visual cortex (V1) and lateral geniculate nucleus (dLGN). Asterisk indicates a significant difference (*, *p* < 0.05). fmj: forceps major corpus callosum; I-VI cortical layer. Scale bars indicate 50 μm (**a**, **b**)

Both NPC patients and *Npc1*^*−/−*^ mice display a prominent dysmyelination, although the mechanism(s) that specifically underlies such loss of myelin are presently not fully understood. In fact, both neuronal and/or oligodendrocyte deficiency of Npc1 protein can severely affect the synthesis of myelin at early postnatal days, resulting in myelin loss with aging [46]. To assess whether a myelin deficit was a putative cause of the increase in the VEP latency of *Npc1*^*−/−*^ mice, brain sections of PN75 sham or HPßCD-treated *Npc1*^*+/+*^ and *Npc1*^*−/−*^ mice were immunostained with an antibody directed against the myelin basic protein (MBP) (Fig. 5). Parasagittal sections encompassing both somatosensory and primary visual cortices (Fig. 5a, b) of *Npc1*^*+/+*^ showed a similar abundance of myelinated fibers in the regions of both sham and HPßCD-treated *Npc1*^*+/+*^, whereas myelinated fibers were below the level of detection in *Npc1*^*−/−*^ mice. MBP immunostainig of optic nerve sections of *Npc1*^*−/−*^ mice also showed a similar pattern of myelin reduction, which was partially rescued by HPßCD administration (Fig. 5c). Moreover, gross histology analyses showed a significant reduction of cellularity in both visual cortex and dLGN (Fig. 6), making it reasonable that a neuronal loss contributed to the visual cortex myelin reduction of *Npc1*^*−/−*^ mice. The cytoarchitecture of visual cortex appeared also disorganized in *Npc1*^*−/−*^ mice, whereas both cellularity and cytoarchitecture were normalized by the HPßCD administration (Fig. 6a).

## Discussion

The rationale for undertaking this study was grounded on accumulating evidence that there is severe neuronal and glial damage in thalamocortical areas of *Npc1*^*−/−*^ mice [15, 47], as well as defective myelin formation and maintenance associated with *Npc1* functional deficiency [46, 48]. These abnormalities are likely associated with an impairment of various sensory pathways, as already indicated by the deficit of auditory [11, 49] and olfactory [50] systems in *Npc1*^*−/−*^ mice and of multisensory processing in NPC patients [51]. As for the visual system of *Npc1*^*−/−*^ mice, retinal cellular and functional defects were recently described [19, 20], but the functional integrity of their visual pathway has not been investigated yet. Now addressing this issue, we report that the transmission of visual stimuli from retina to visual cortex is strongly influenced by the lack of *Npc1* function. In fact, compared to naïve *Npc1*^*+/+*^ mice, naïve *Npc1*^*−/−*^ mice displayed a significant increase in the latency and a dramatic decrease in the amplitude of their N1-N3 VEP components, likely due to gross histological and cellular alterations of the visual system of these mice, including cholesterol accumulation, reduced cellularity, and a high level of demyelinization along the entire visual stimulus itinerary, from retina to visual cortex (present data, [46, 52]).

Different cellular/functional mechanism(s) are likely underlying the VEP features of sham *Npc1*^*+/+*^ and *Npc1*^*−/−*^ mice. In the case of *Npc1*^*+/+*^ mice, in fact, the postnatal injection of plain PBS elicited dramatic VEP changes during adulthood resulting in a significant decrease of both latency and amplitude. These modifications are likely to reflect the ability of normal mouse brain to modify its structure and function in response to a week and transitory stress(es) (as the interscapular injections, transient body constriction and brief maternal separation performed in our experiments) during early postnatal life. Among these putative stressors, maternal separation should be ruled out because its duration in present experiments (20 min) was much shorter than the 3 h duration required for a permanent negative effect during adulthood in the mouse [53]. In contrast, it is well documented that the administration of short-lasting stresses during infancy elicits an opposite effect, increasing adult brain resilience to stress in both mice and humans [54]. In line with this view, the significant decrease in VEP latency and amplitude of sham *Npc1*^*+/+*^ mice suggests that, among other possibilities, the early postnatal stress improved the adult visual pathway efficiency and specialization (decrease in latency) and reduced the overall number of stimulus-locked oscillations (decrease in amplitude) [55].

While the VEP response of sham *Npc1*^*+/+*^ mice is apparently modified by the postnatal stress, this is likely not true for sham *Npc1*^*−/−*^ mice. In this case, in fact, the injection protocol did not elicit any significant effect on both VEP latency and amplitude, suggesting these mice were actually unable to respond to postnatal stress as wild-type mice did. We favor the hypothesis that such ability was missing in *Npc1*^*−/−*^ mice because of the severe structural and/or functional disorganization of their visual system, including eye, optic nerve, dLGN and cortex. It is also worth to note that, although the small VEP amplitude of naïve/sham *Npc1*^*−/−*^ mice was statistically similar to that of sham *Npc1*^*+/+*^ mice, it likely was very different in nature. In fact *Npc1*^*−/−*^ sham mice also displayed an abnormally high value of VEP latency, in line with their altered neuroanatomical features.

The latency value of HPßCD-treated *Npc1*^*+/+*^ mice was similar to that of *Npc1*^*+/+*^ sham mice, indicating uneffectiveness of the drug. Indeed, both HPßCD-treated and sham *Npc1*^*+/+*^ mice displayed an approximately 3 msec reduction of N1 latency compared to that of *Npc1*^*+/+*^ naive mice, leading to the conclusion that the latency decrease of *Npc1*^*+/+*^ sham and HPßCD-treated mice is a consequence of the stress elicited by pup manipulation associated with the injection *per se*. This conclusion is in agreement with a previous study showing a similar (3–3.5 msec) reduction in VEP latency of adult mice that had received repeated saline injections and manipulation-elicited stress during early postnatal life [56].

The response of *Npc1*^*−/−*^ mice to HPßCD administration was completely different. In fact the drug rescued the increased VEP latency of *Npc1*^*−/−*^ mice, eliciting latency values that nicely approximated those of both sham and HPßCD-treated *Npc1*^*+/+*^ mice. This beneficial effect was therefore associated with the drug rather than with the injection/manipulation *per se*.

Both sham and HPßCD treatments did not determine any significant difference between the amplitudes of *Npc1*^*+/+*^ and *Npc1*^*−/−*^ mice. High variability and low consistency are frequently observed in repeated amplitude recordings of the same animal (not shown), likely because this component is greatly influenced by the background level of ongoing cortical activity [57, 58]. For this reason, latency is generally considered a more robust and reliable indicator of the visual pathway integrity [52] compared to amplitude. This is particularly true for a mouse model presenting demyelination as the *Npc1*^*−/−*^ mouse [46], in which the latency delay we report in this study likely reflects, among other cellular/histological alterations, the high level of demyelination along the visual stimulus itinerary [52].

By unveiling the different VEP response of sham and HPßCD-treated *Npc1*^*+/+*^ and *Npc1*^*−/−*^ mice, our results further strengthen the specific effect of HPßCD treatment in normalizing *Npc1*^*−/−*^ brain structural/functional organization, including that of visual pathway. Accordingly, we show that cholesterol accumulation in retinal ganglion cells, in dLGN neurons and in visual cortex are largely rescued by the HPßCD administration, which also counteracts the robust myelin reduction in optic nerve fibers and axons projecting to the visual cortex of *Npc1*^*−/−*^ mice.

## Conclusions

The results of this study based on VEP analysis expand recent observations on the presence of severe neuronal and glial damage in brain areas as the thalamus, correlating these features to the impairment of visual stimulus transmission from retina to visual cortex. They also provide an additional insight on the effectiveness and safety of HPßCD administration. In light of these findings, VEP analysis appears as a putatively accurate and non-invasive tool to monitor neural activity and sensory processing in NPC1 patients, as well as to monitor disease progression and to assess the efficacy of potential therapies.

## Additional files

Additional file 1: Figure S1. A schematic summary of sham/ HPßCD injections, electrode implantation and VEP recording in various experimental groups. (JPEG 2651 kb) Additional file 2: Figure S2. Survival curves of naive *Npc1* ^*−/−*^, sham *Npc1* ^*−/−*^ and HPβCD *Npc1* ^*−/−*^mice. Kaplan-Meier plots were analyzed by Log-rank (Mantel -Cox) test: *Npc1* ^*−/−*^ vs *Npc1* ^*−/−*^ sham, *p* > 0.05; *Npc1* ^*−/−*^ vs *Npc1* ^*−/−*^ HPβCD, *p* < 0.0001. Survival (mean value ± SEM): naive *Npc1* ^*−/−*^, 91.3 ± 1.3; sham *Npc1* ^*−/−*^, 87.8 ± 0.9; HPβCD *Npc1* ^*−/−*^, 145 ± 3.0. (JPEG 2830 kb) Additional file 3: Figure S3. Circles indicate the brain areas selected for histological analyses. 1, primary visual cortex; 2, dorso lateral geniculate nucleus; 3, retina. V1, primary visual cortex; dLGN, dorso lateral geniculate nucleus; CPu, caudate putamen; cc, corpus callosum; LV, lateral ventricle; S1, primary somatosensory cortex. (JPEG 3262 kb) Additional file 4: Table S1. A comparison of VEP N1 latency and amplitude of PN75 naive *Npc1* ^*+/+*^ *,* sham *Npc1* ^*+/+*^ *,* naive *Npc1* ^*−/−*^ and sham *Npc1* ^*−/−*^. (JPEG 2961 kb) Additional file 5: Table S2. Effect of age and disease progression (PN60-PN100) on VEP N1 latency. (JPEG 2726 kb) Additional file 6: Table S3. VEP latency and amplitude in PN60, PN75 and PN85, either sham or HPβCD -treated, *Npc1* ^*+/+*^ and *Npc1* ^*−/−*^ mice. (JPEG 2776 kb) Additional file 7: Table S4. VEP latency and amplitude of either sham or HPβCD -treated PN100 *Npc1* ^+/+^ and *Npc1* ^−/−^ mice. (JPEG 2673 kb)

## Abbreviations

CPu: Caudate putamen
dLGN: Dorsal lateral geniculate nucleus
ERG: Electroretinogram
Fmj: Forceps major corpus callosum
GCL: Ganglion cell layer
HPßCD: 2-hydroxypropyl-ß-cyclodextrin
INL: Inner nuclear layer
IPL: Inner plexiform layer
MBP: Myelin basic protein
MGN: Medial geniculate nucleus
NPC1: Niemann Pick type C1
ONL: Outer nuclear layer
OPL: Outer plexiform layer
OS: Outer segments
PFA: Paraformaldehyde
PN: Post natal day
ROI: Region of interest
RPE: Retinal pigment ephitelium
V1: Primary visual cortex
VEP: Visual evoked potential
VPL: Ventral postero-lateral
VPM: Ventral postero-medial
