# Supplementary figures and images for: Visual evoked potentials of Niemann-Pick type C1 mice reveal an impairment of the visual pathway that is rescued by 2-hydroxypropyl-ß-cyclodextrin
Source: Orphanet J Rare Dis. 2015 Oct 12;10:133. doi: 10.1186/s13023-015-0348-0 (PMC4603821; doi:10.1186/s13023-015-0348-0)

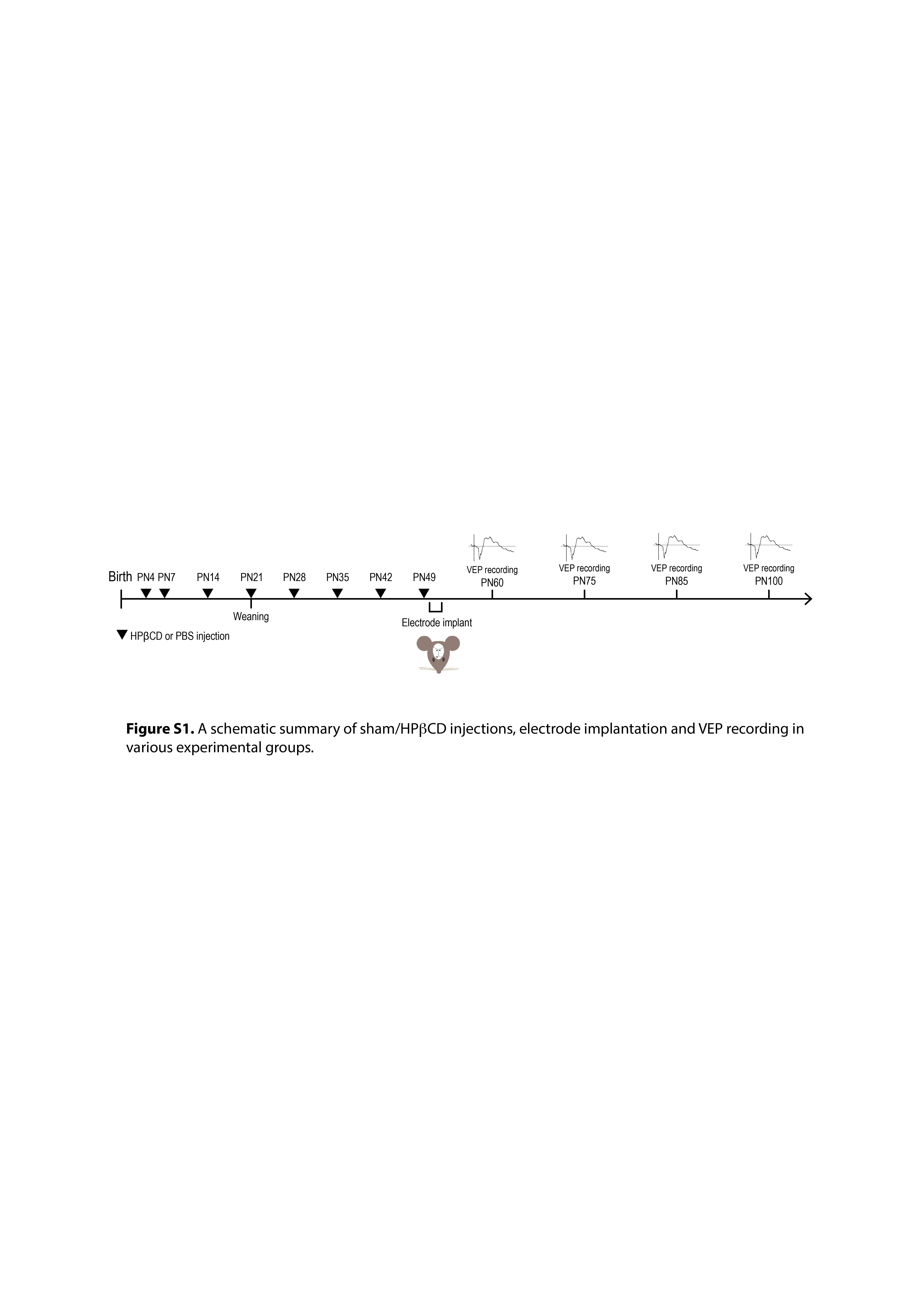

Supplement: Additional file 1: Figure S1. — A schematic summary of sham/ HPßCD injections, electrode implantation and VEP recording in various experimental groups. (JPEG 2651 kb) [file 13023_2015_348_MOESM1_ESM.jpg]

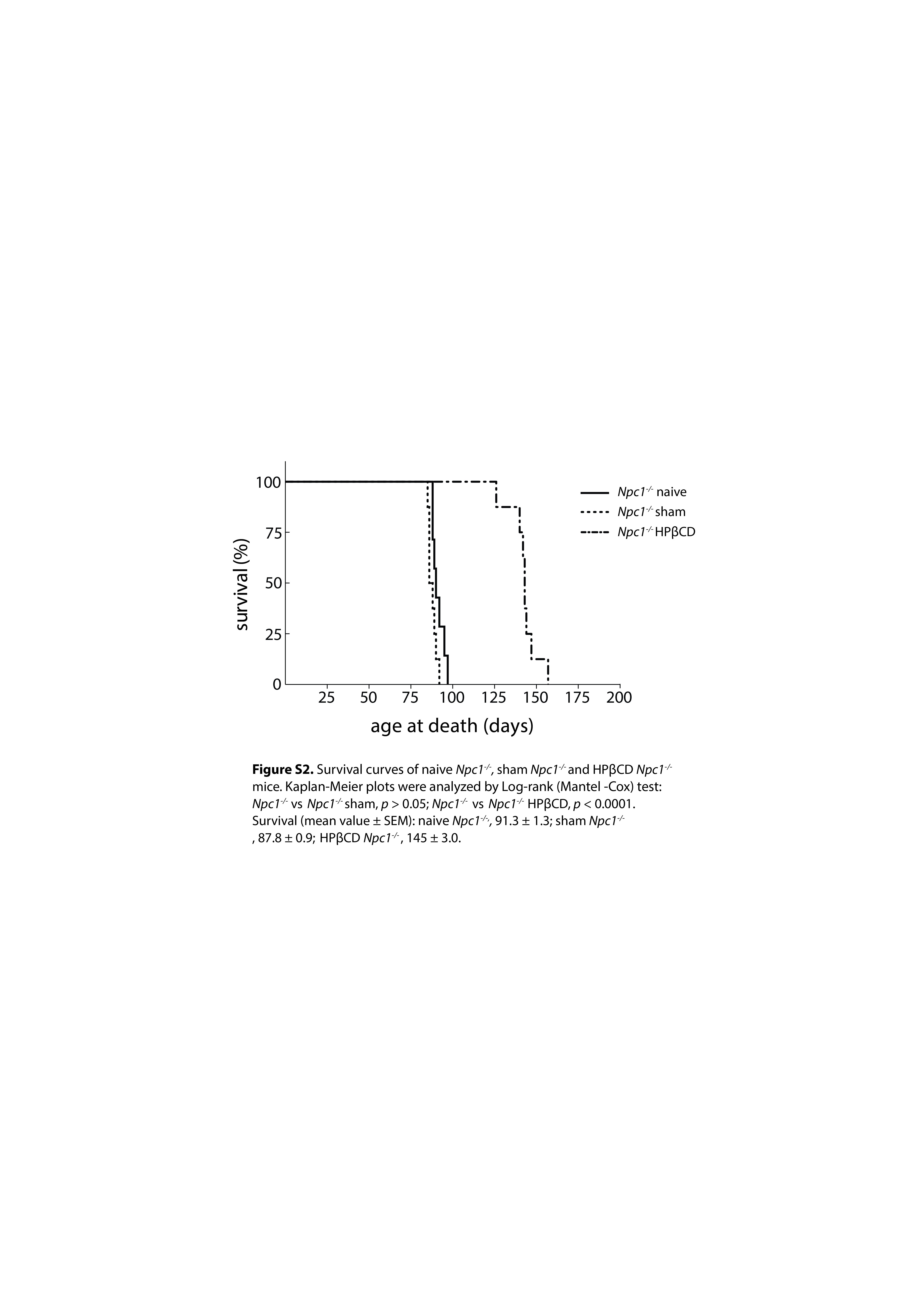

Supplement: Additional file 2: Figure S2. — Survival curves of naive Npc1 −/−, sham Npc1 −/− and HPβCD Npc1 −/−mice. Kaplan-Meier plots were analyzed by Log-rank (Mantel -Cox) test: Npc1 −/− vs Npc1 −/− sham, p > 0.05; Npc1 −/− vs Npc1 −/− HPβCD, p < 0.0001. Survival (mean value ± SEM): naive Npc1 −/−, 91.3 ± 1.3; sham Npc1 −/−, 87.8 ± 0.9; HPβCD Npc1 −/−, 145 ± 3.0. (JPEG 2830 kb) [file 13023_2015_348_MOESM2_ESM.jpg]

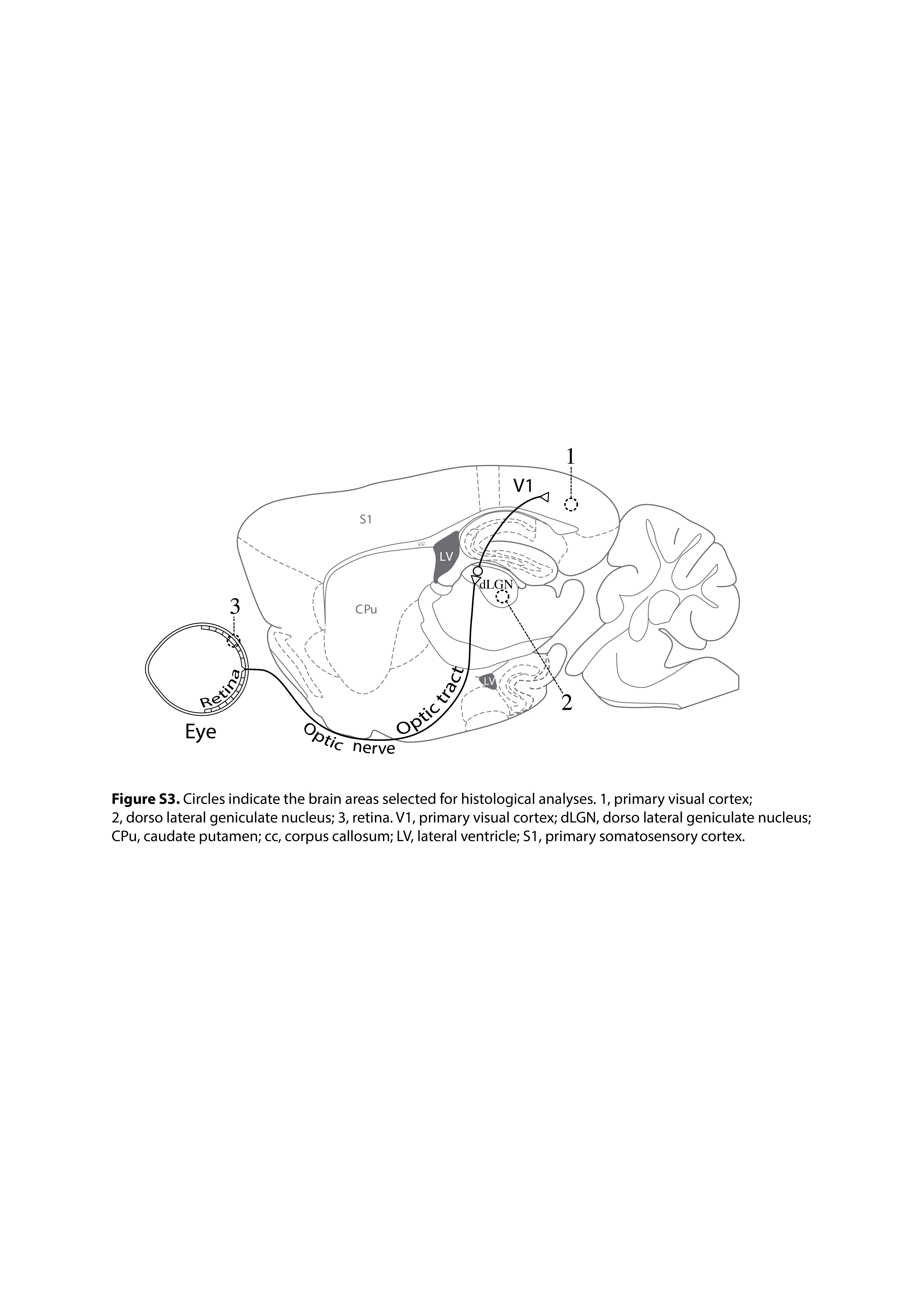

Supplement: Additional file 3: Figure S3. — Circles indicate the brain areas selected for histological analyses. 1, primary visual cortex; 2, dorso lateral geniculate nucleus; 3, retina. V1, primary visual cortex; dLGN, dorso lateral geniculate nucleus; CPu, caudate putamen; cc, corpus callosum; LV, lateral ventricle; S1, primary somatosensory cortex. (JPEG 3262 kb) [file 13023_2015_348_MOESM3_ESM.jpg]

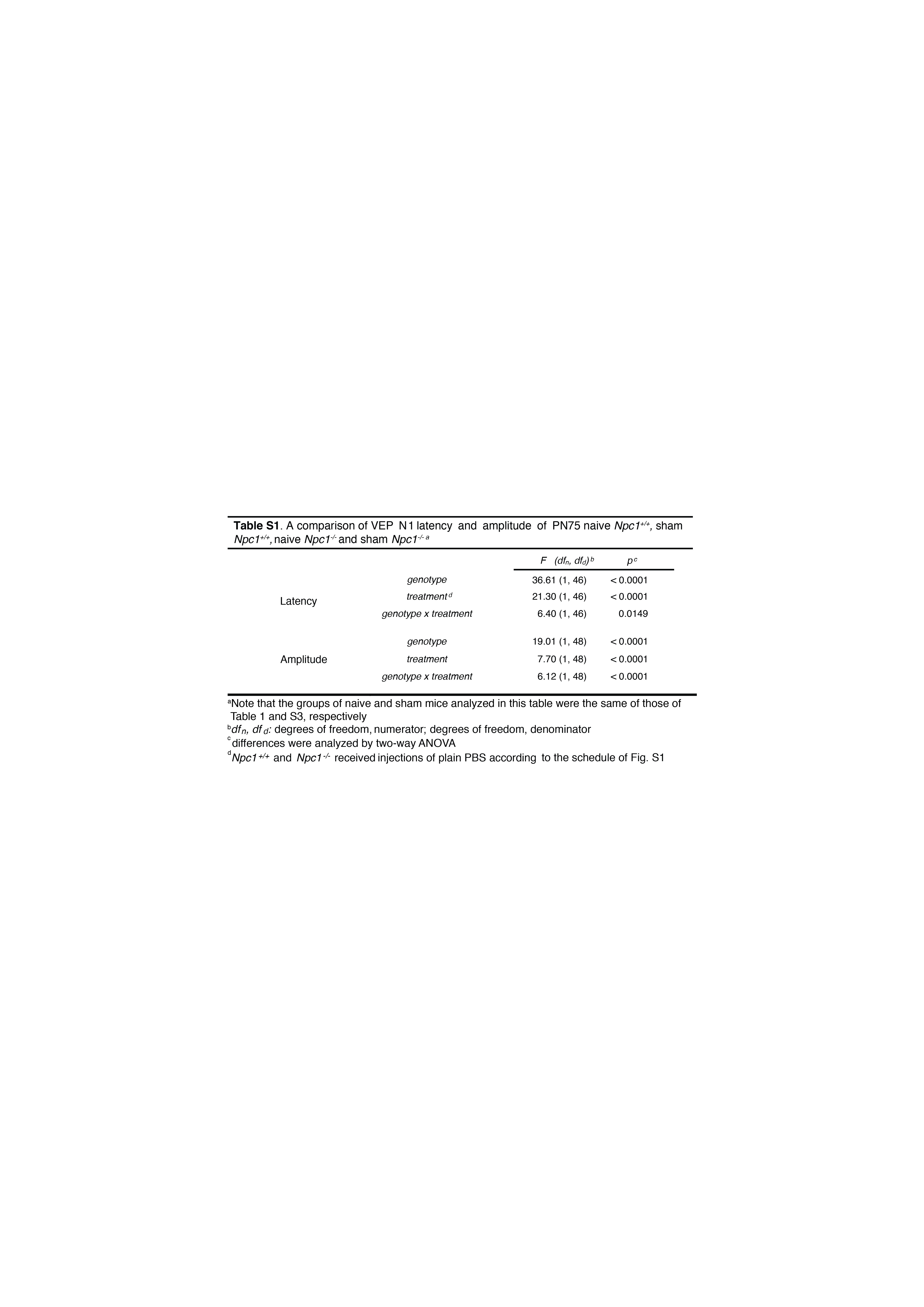

Supplement: Additional file 4: Table S1. — A comparison of VEP N1 latency and amplitude of PN75 naive Npc1 +/+ , sham Npc1 +/+ , naive Npc1 −/− and sham Npc1 −/−. (JPEG 2961 kb) [file 13023_2015_348_MOESM4_ESM.jpg]

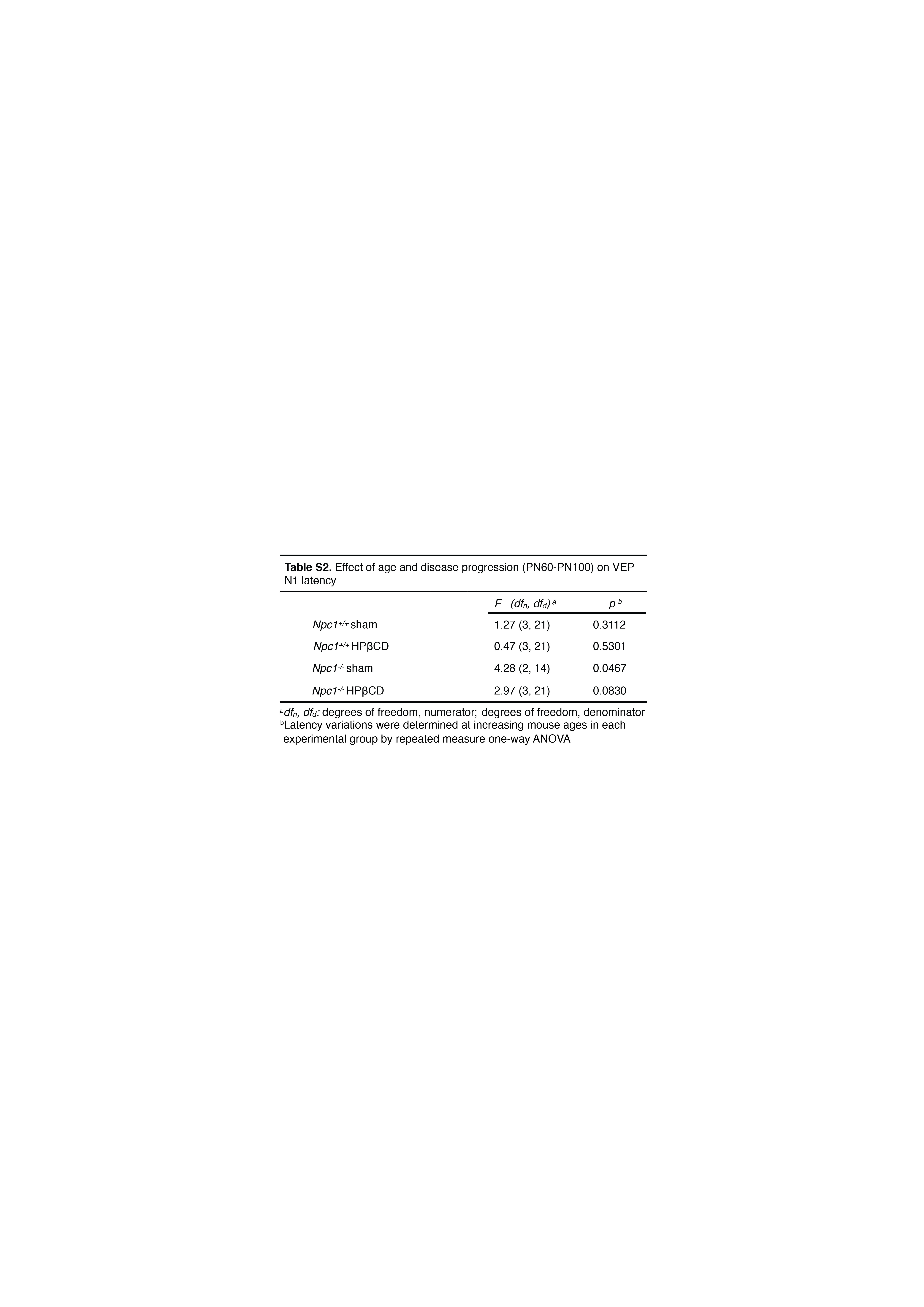

Supplement: Additional file 5: Table S2. — Effect of age and disease progression (PN60-PN100) on VEP N1 latency. (JPEG 2726 kb) [file 13023_2015_348_MOESM5_ESM.jpg]

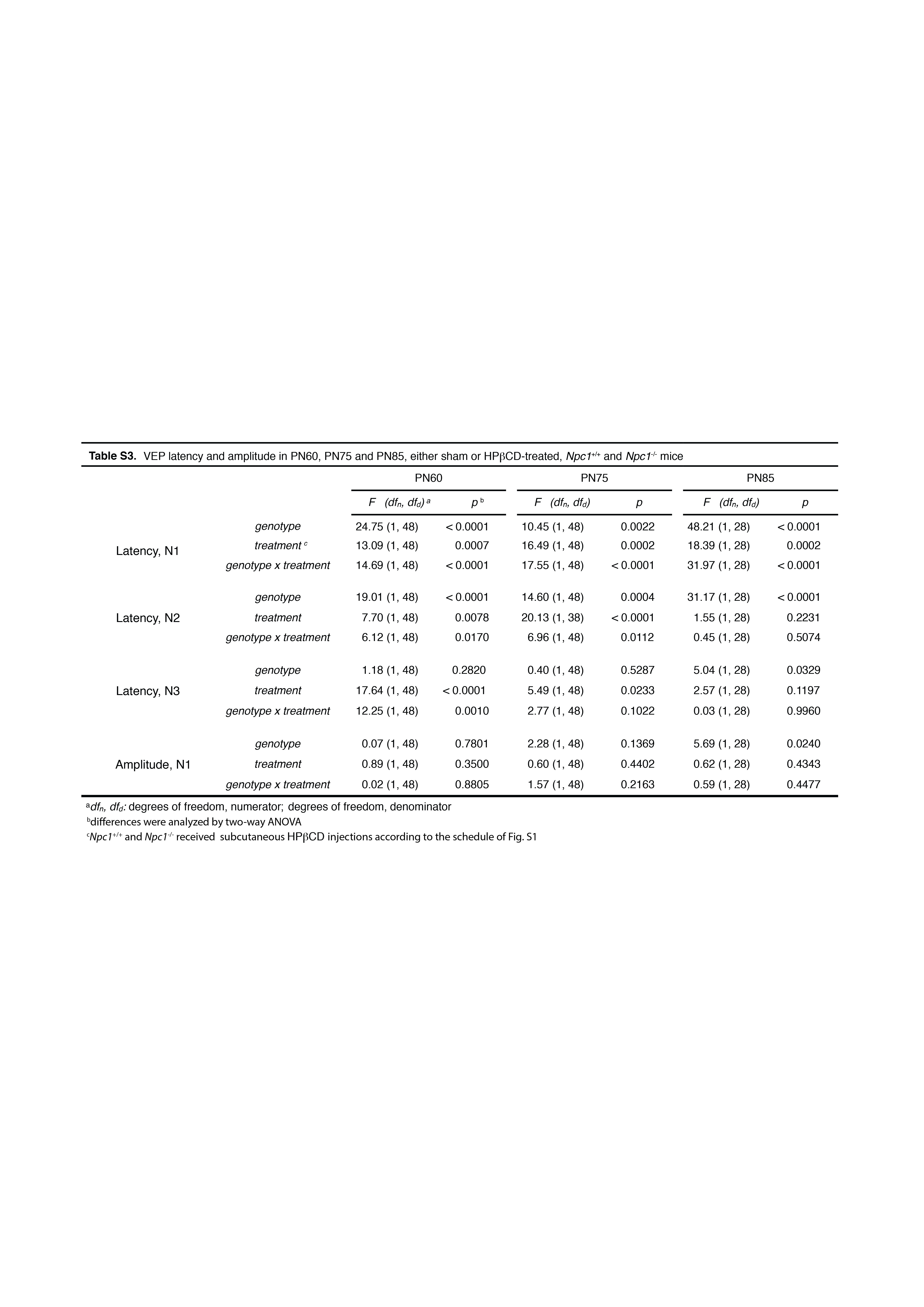

Supplement: Additional file 6: Table S3. — VEP latency and amplitude in PN60, PN75 and PN85, either sham or HPβCD -treated, Npc1 +/+ and Npc1 −/− mice. (JPEG 2776 kb) [file 13023_2015_348_MOESM6_ESM.jpg]

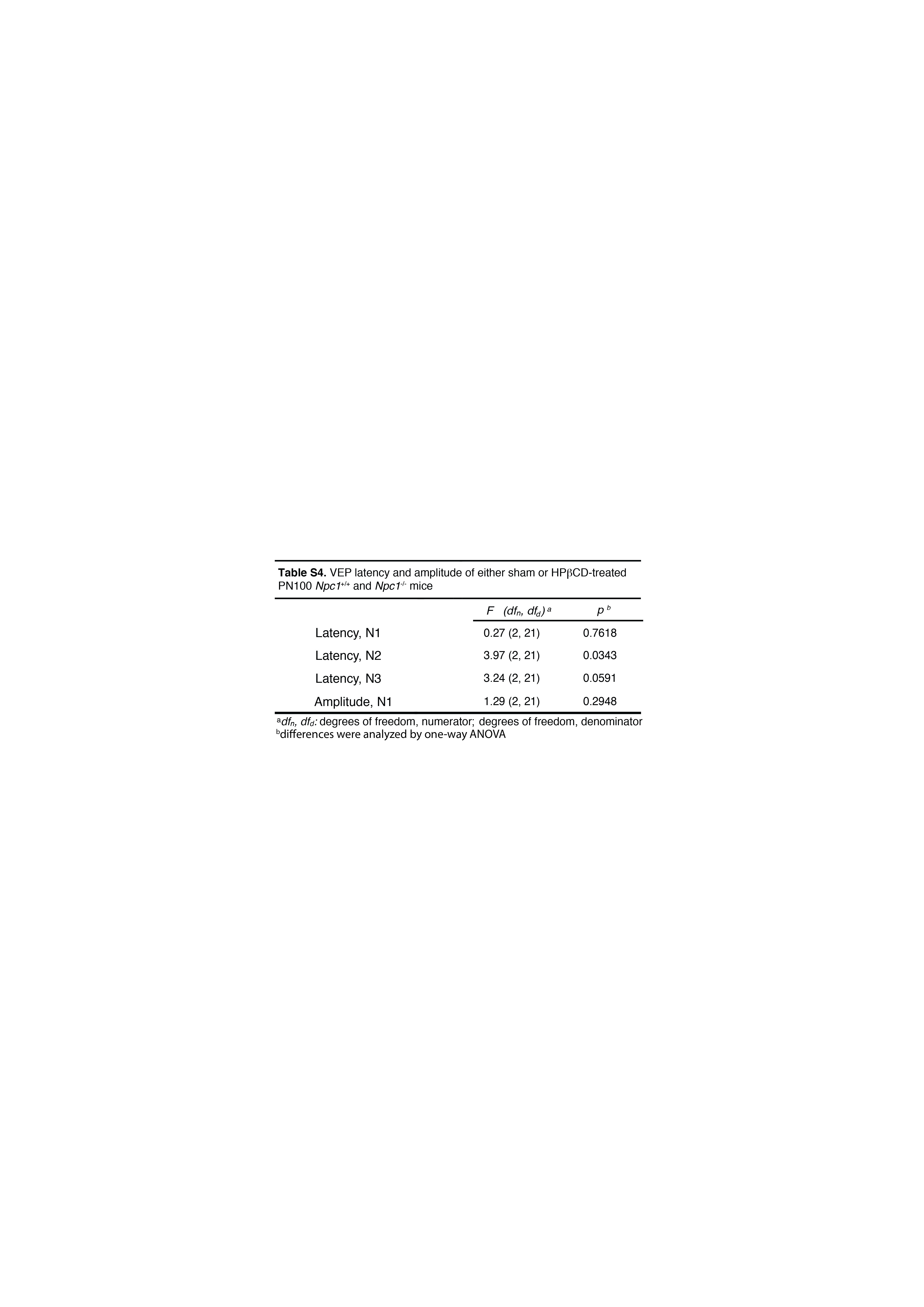

Supplement: Additional file 7: Table S4. — VEP latency and amplitude of either sham or HPβCD -treated PN100 Npc1 +/+ and Npc1 −/− mice. (JPEG 2673 kb) [file 13023_2015_348_MOESM7_ESM.jpg]
